# Method for correction of experimental action spectrum using actual overlapping spectra of radiation sources

**DOI:** 10.1016/j.mex.2021.101221

**Published:** 2021-01-07

**Authors:** Mikhail Lyulyukin, Nikita Kovalevskiy, Sofya Timofeeva, Egor Gusachenko, Maria Solovyeva, Dmitry Selishchev, Denis Kozlov

**Affiliations:** a Novosibirsk State Technical University, Novosibirsk 630073, Russia; b Boreskov Institute of Catalysis, Novosibirsk 630090, Russia; c Novosibirsk State University, Novosibirsk 630090, Russia

**Keywords:** Photoresponse, Irradiation, Spectra, Basic wavelength, Action spectra

## Abstract

Experimental dependency of the photosystem's response on the wavelength of exciting radiation, also known as action spectrum, may be substantially affected by the spectrum shape of this radiation. This is especially important in the case, when different radiation sources are used for the investigation of action spectrum. For instance, too wide emission peaks of radiation sources can blur the scopes of actual action spectrum and distort information about the properties of photosystem at certain wavelength regions. Here, we propose a method for the correction of experimental action spectrum by the recalculation of experimental data of photoresponse according to actual spectra of exciting radiation. In the case of overlapping radiation spectra from different radiation sources, this method results in much better correlation of experimental action spectrum to actual action spectrum or absorption spectrum of photosystem. The data on photoactivity of several photocatalysts are presented to illustrate and validate the proposed method.

• Activity of photosystem depends on the actual spectrum of the radiation source
• Single-peak optical radiation sources with the same basic wavelength may cause a different photoactivity
• Effect of actual spectrum of the light source on the photoactivity is to be considered

## Graphical abstract

**Figure:**
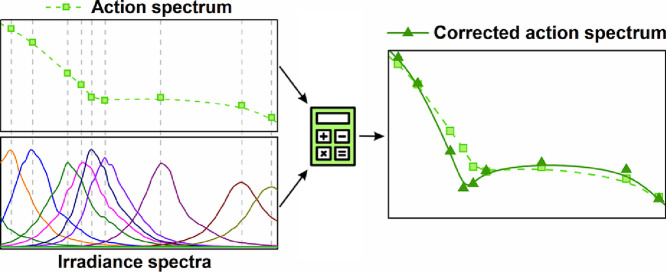

Specifications table

**Table:** 

Subject Area:	Chemistry
More specific subject area:	Photochemistry and photocatalysis
Method name:	Method for correction of action spectrum using irradiation spectra (MCASIS)

## Method details

### Background

High-resolution action spectra are of great use in photochemistry because they can help to predict the response of photosystem under certain irradiation. High resolution can only be achieved by using light sources with a narrow spectral distribution of photons. Therefore, the most common approach nowadays is using of a powerful broadband light source combined with a monochromator. This way, Xe-lamp with monochromator was reported as the excitation source in research papers [1], [2], [3], and several articles with presented values of full width at half maximum (FWHM) for spectra of used irradiations in nm are presented in tutorial review paper [4]. In the modern world, LED light sources, which are more energy-efficient and easier to use than large broadband light sources, are frequently used. However, their spectra are not perfect, and they have an FWHM significantly exceeding units of nanometers - from 10 up to 75 nm [5]. This may cause blurring of spectra and distortion of important data.

The analysis of the action spectra of catalytic systems obtained using seventeen LED light sources with different basic wavelengths and the analysis of their emission spectra overlapping gave us the motivation to create a method for the correction of experimental action spectra of photocatalytic systems. The essence of the method is finding of a solution for the inverse problem: to construct an actual action spectrum for the system, which will give the experimentally observed activity under the specific radiation from the selected light sources.

The values of incident photon flux $q_{n,p}^{0}$ (mol s^−1^ or, which is the same for photons, E s^−1^) for light sources (or specific radiations, obtained from a single broadband light source using various optical devices: filter, multipliers, monochromators, etc.) may be measured with photon counters or recalculated from the data obtained using spectroradiometer, as follows:

(1) $$q_{n,p}^{0}=S\int_{\lambda_{1}}^{\lambda_{2}}E_{n,p,\lambda }^{0}d\lambda ,$$

where *S* is the geometric surface area of irradiated photocatalyst, $E_{n,p,\lambda }^{0}$ is the spectral photon irradiance (E s^−1^ cm^−2^ nm^−1^).

The discrete experimental data points of activity, $a_{n,i}$ (mol s^−1^), can be expressed as a function of the actual action spectrum ($A_{\lambda }$), and spectral photon irradiance of photocatalyst under each radiation as follows:

(2) $$a_{n,i}=S\int_{\lambda_{\min}}^{\lambda_{\max}}A_{\lambda }E_{n,p,\lambda ,i}^{0}d\lambda ,$$

where $a_{n,i}$ (mol s^−1^) is the photocatalytic activity detected under *i*^th^ radiation, $\lambda_{\min}$ and $\lambda_{\max}$ (nm) correspond to the edges of irradiance peak for the considered optical radiation source, $E_{n,p,\lambda ,i}^{0}$ (E s^−1^ cm^−2^ nm^−1^) is the spectral photon irradiance of catalyst surface under *i*^th^ radiation source, and $A_{\lambda }$ (mol E^−1^, i.e. dimensionless) is the efficiency of photon utilization at a certain wavelength.

### Algorithm steps

1. As an initial approximation of the actual action spectrum of the catalyst, the experimentally obtained dependence of photonic efficiency on the wavelength should be taken with the assumption of the monochromaticity of the incident light.
2. Since the experimentally observed values are discrete points, to obtain a continuous function, the points are connected by linear segments that interpolate the values of the catalyst photoresponse within the full range of irradiations used. The use of nonlinear functions (for example, spline interpolation) may give a greater gain in the quality of the resulting approximations. But using “curved” approximation functions should be avoided if no reason for the opposite exists.
3. For each wavelength corresponding to the basic wavelength of single radiation, the integral value of (2) is numerically calculated. This results in an array of values corresponding to the integral of product of the photocatalyst photonic efficiency spectrum and the incident radiation spectrum.
4. Then, the obtained integral value for each wavelength is compared with the experimentally observed photonic efficiency value under the irradiance with a peak position at the indicated wavelength. If the integral value (2) for the specified wavelength exceeds the experimental discrete point – the action spectrum point is reduced by a delta value (selected based on the conditions, e.g. about 10% of the difference between experimental and integrated values) in the next iteration step, if less – the spectrum point is increased by this delta value in the next iteration step.
5. As a numerical estimate of the proximity of the calculated action spectrum to the actual one, the sum of the squares of the difference between the values of the corrected spectrum and the experimental activity values observed under LEDs with the corresponding radiation peak is used.
6. Then, steps 1–5 are repeated until the resulting value of the sum of squares of differences is stabilized. This means that the correction of action spectrum was made with maximum reachable accuracy.

This algorithm can be implemented using any available programming language, and its implementation does not require a great computational resource.

### Method validation

**Fig. 1:**
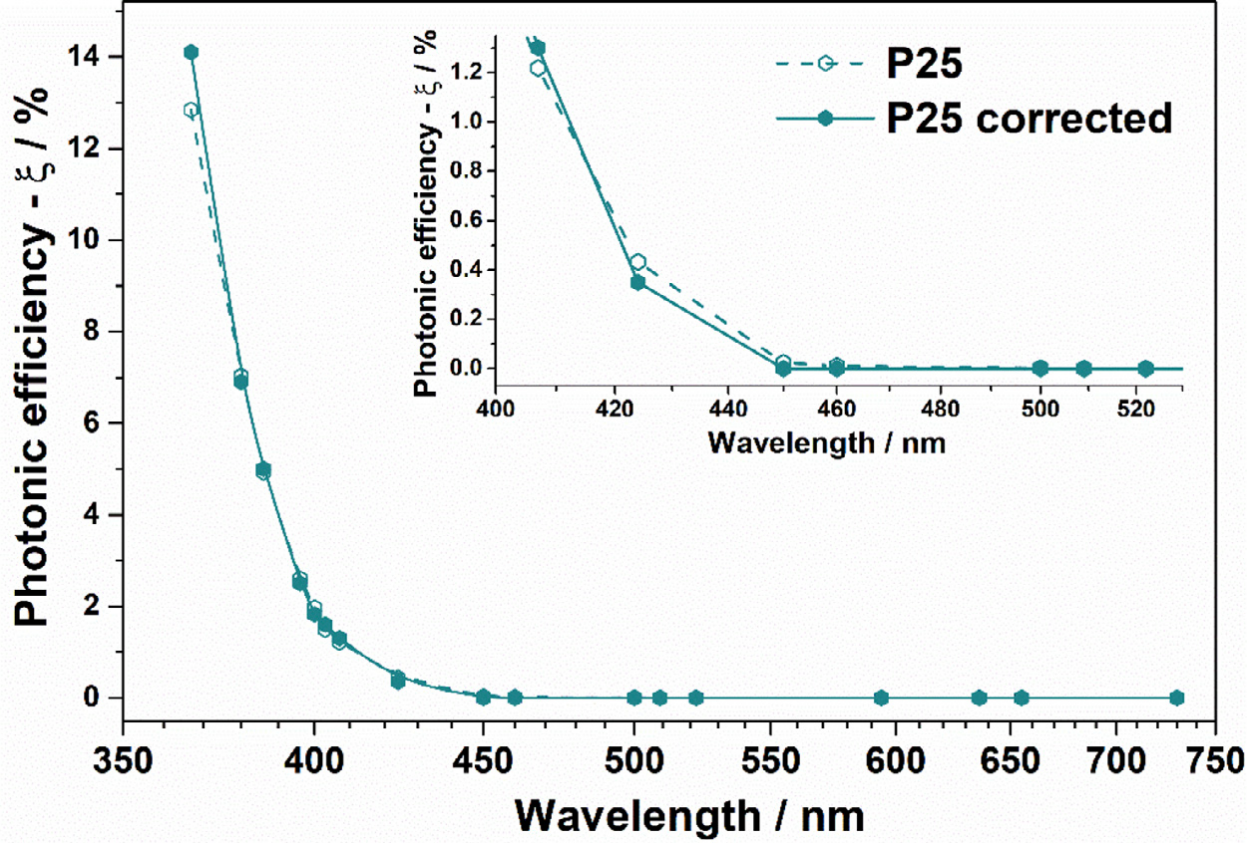
Experimental and corrected action spectra of Evonik P25 for the photocatalytic oxidation (PCO) of acetone.

The proposed method was checked for the correction of experimentally obtained action spectra of several photocatalysts using 17 single-peak LEDs with FWHM from 11 to 40 nm. The implementation of the method for correction of the action spectra was found to be crucial for the samples with a sharp transition from an area with activity to an area without observed activity. For example, for a sample of TiO_2_ Evonik P25 photocatalyst, without light absorption for wavelengths >420 nm, no photoactivity should be observed in the specified region. But the observed activity indicates the existence of response of P25 to the radiation from LED with basic wavelength of 460 nm. The experimentally obtained data on the dependence of the photonic efficiency (PE) on the wavelength for P25 are presented in Table 1, along with the corrected photonic efficiency value (CPE), the integral value of the expression (2) (IPEE), and the difference between the experimental and integral values for each wavelength. Graphical illustration of the initial and corrected spectra is shown in Fig. 1.

**Table 1:** Recalculation data for correction of P25 action spectra.

Basic wavelength, nm	Photonic efficiency (PE), %	Corrected photonic efficiency (CPE), %	Integral product of efficiency and emission (IPEE), %	Difference (PE)-(IPEE), %
367	12.843	14.1	12.864	-0.021
380	7.032	6.9	7.026	0.0006
386	4.926	5.05	4.932	-0.0006
396	2.596	2.5	1.938	0.66
400	1.967	1.82	2.584	-0.62
403	1.492	1.6	1.495	-0.003
407	1.217	1.3	1.227	-0.01
424	0.433	0.35	0.42	0.013
450	0.023	0	0.032	0.009
460	0.011	0	0.009	0.002
500	0	0	0	0
512	0	0	0	0
526	0	0	0	0
593	0	0	0	0
634	0	0	0	0
654	0	0	0	0
734	0	0	0	0

It is seen from the presented data that after the correction, the observed endpoint (460 nm) of the activity region is changed, and the actual value of the endpoint (424 nm) better correlates with the known data on the optical properties of the P25.

**Fig. 2:**
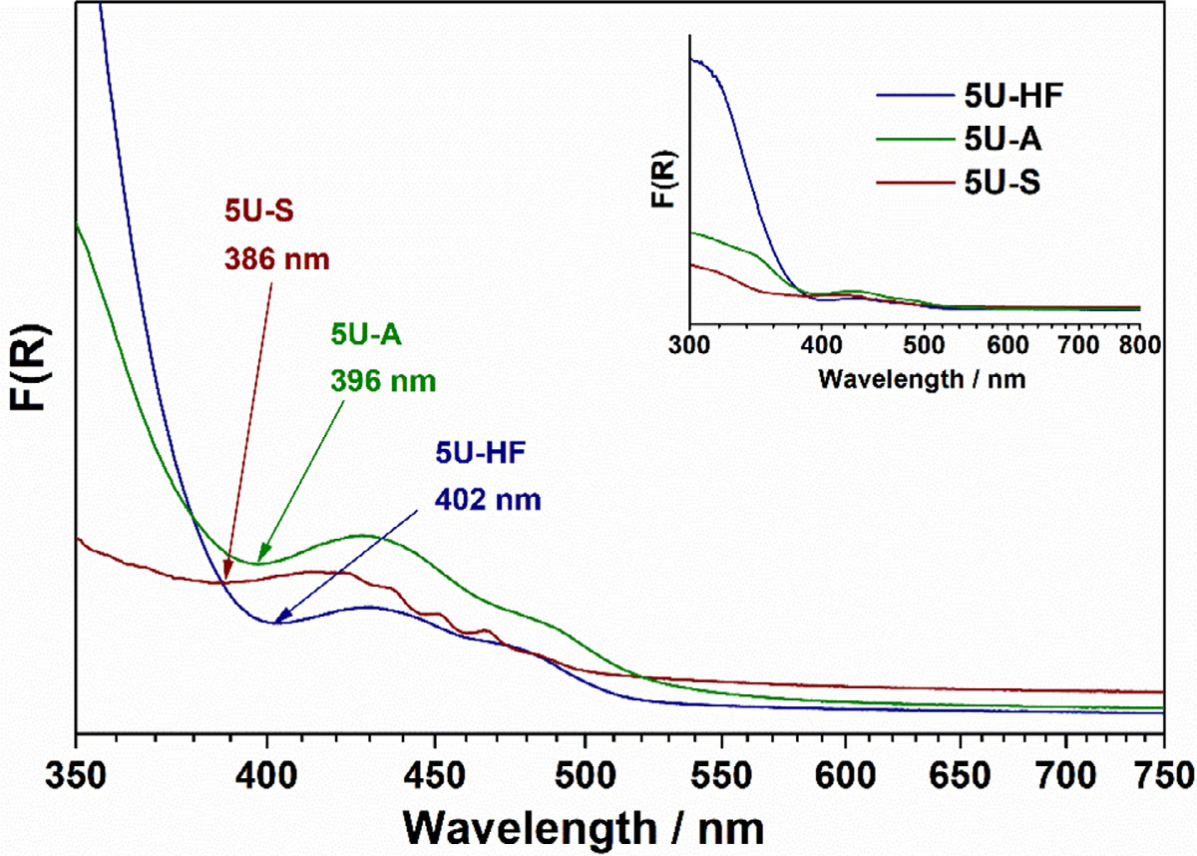
UV–vis spectra for 5U-A, 5U-S, and 5U-HF samples.

**Fig. 3:**
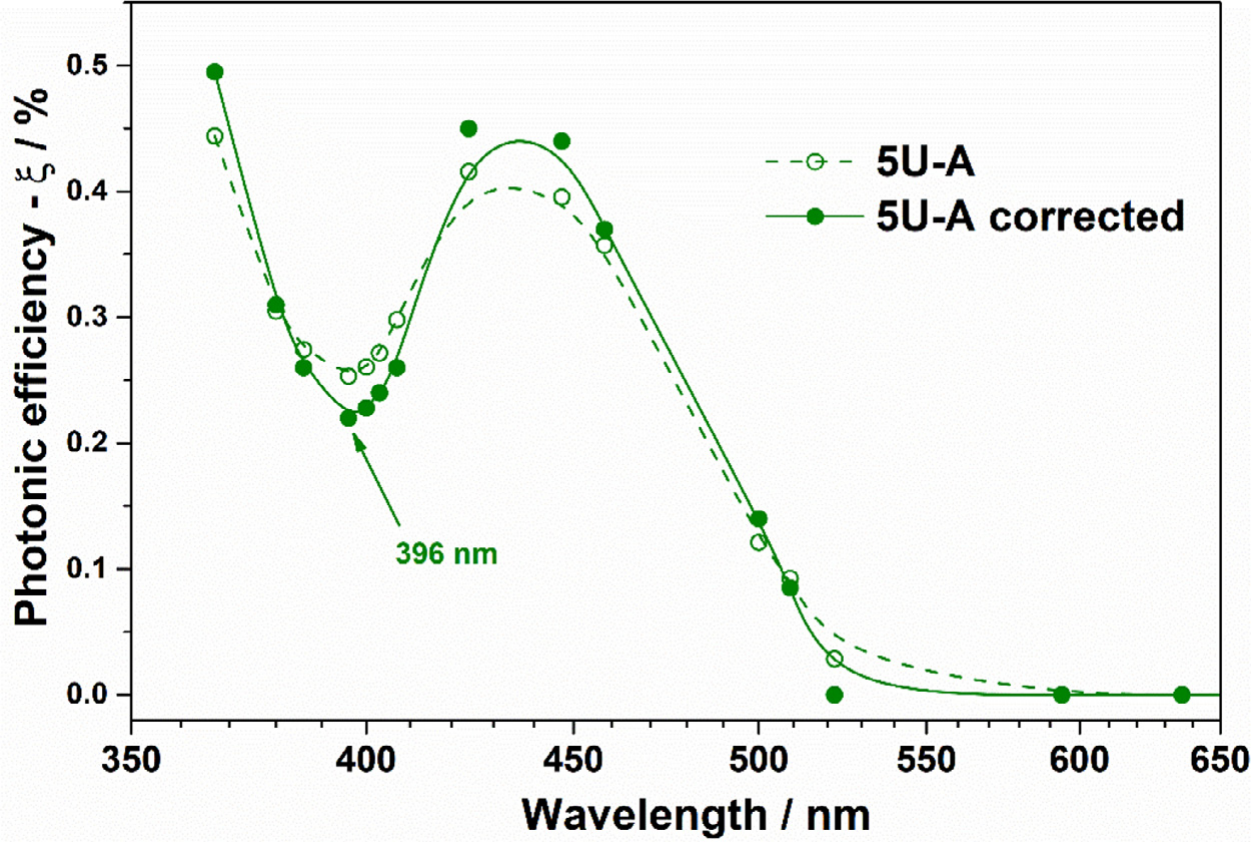
Experimental and corrected action spectra of uranyl-modified Al_2_O_3_ (5U-A) for the PCO of acetone.

**Fig. 4:**
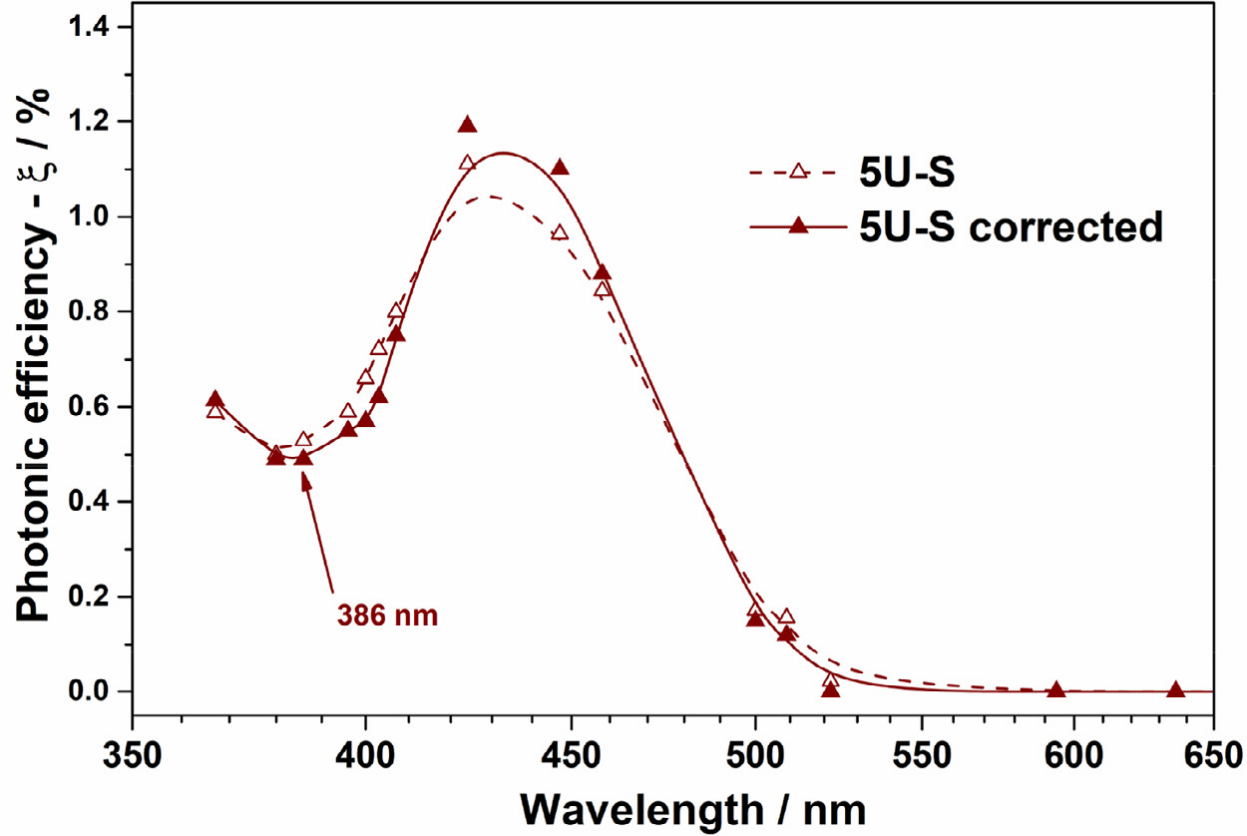
Experimental and corrected action spectra of uranyl-modified SiO_2_ (5U-S) for the PCO of acetone.

**Fig. 5:**
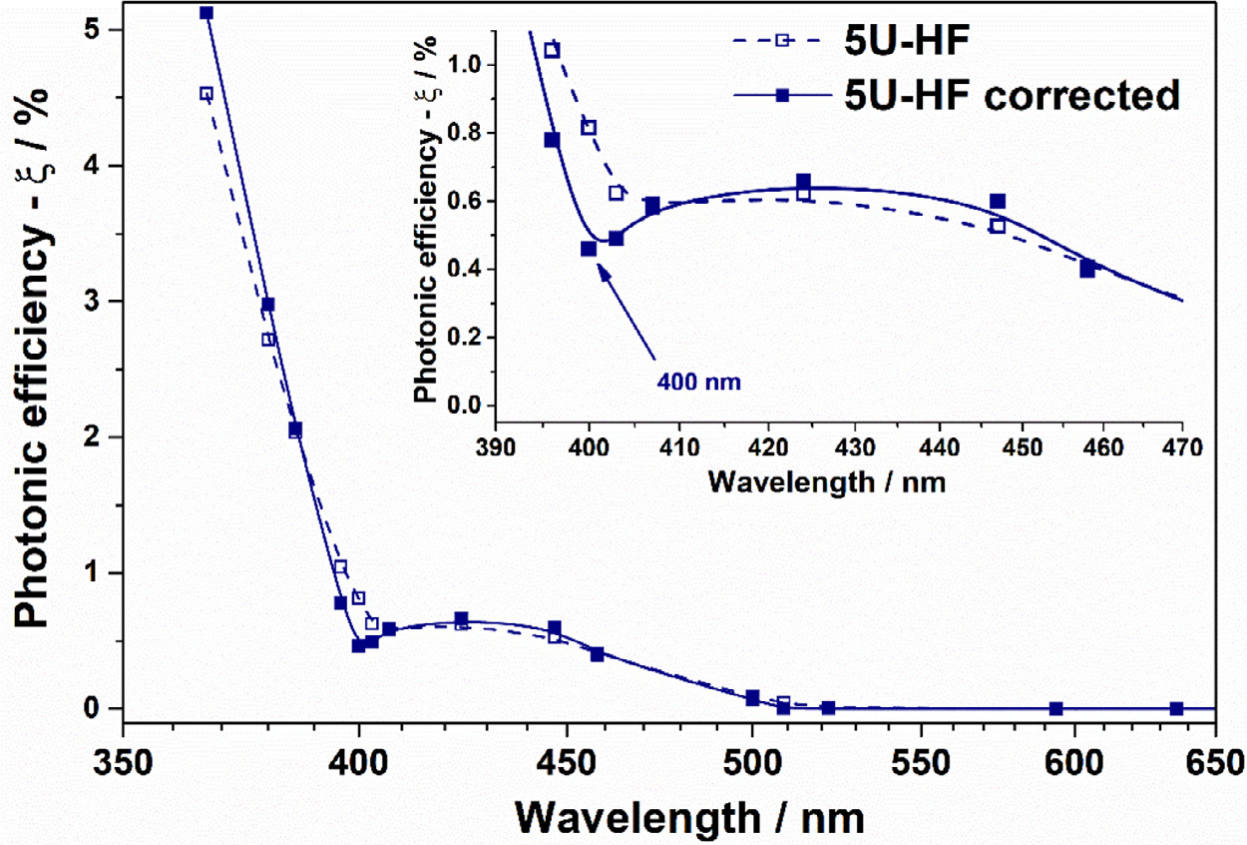
Experimental and corrected action spectra of uranyl-modified TiO_2_ (5U-HF) for the PCO of acetone.

For the illustration of method implementation for action spectra with several points of local maxima and minima of activity, we selected samples 5U-A, 5U-S, and 5U-HF, which are uranyl-modified Al_2_O_3_, SiO_2_, and TiO_2_, respectively. The details of the synthesis and characterization of the samples can be found elsewhere [6], [7], [8]. UV–Vis diffuse reflectance spectra for these samples recorded using a Cary 300 UV–Vis spectrophotometer from Agilent (USA) equipped with a DRA-30I diffuse reflectance accessory are shown in Fig. 2. Experimental and corrected action spectra for 5U-A, 5U-S, and 5U-HF are shown in Figs. 3–5, respectively.

It should be noted that the position of a local minimum of activity has to be in correlation with the position of light absorbance minimum. Therefore, the minimum of activity for the modified alumina (Fig. 3) remained unchanged after the correction, but the endpoint of activity moved to the shorter wavelength in the same way, as it was for P25.

For the modified silica (Fig. 4), not only the endpoint of the activation region had changed, but also the position of a local minimum of activity moved to 386 nm, which better correlates with UV–vis data than the uncorrected spectra.

The most noticeable change was observed for the modified titania (Fig. 5): the experimental action spectra had a “step”, but the local minimum at the wavelength of 400 nm appeared after the correction, which better correlates with UV–vis data.

## Declaration of Competing Interest

The Authors confirm that there are no conflicts of interest.
